# Unmanned Aerial System-Based Data Ferrying over a Sensor Node Station Network in Maize

**DOI:** 10.3390/s22051863

**Published:** 2022-02-26

**Authors:** Jasreman Singh, Yufeng Ge, Derek M. Heeren, Elizabeth Walter-Shea, Christopher M. U. Neale, Suat Irmak, Mitchell S. Maguire

**Affiliations:** 1 Department of Biomedical, Biological and Chemical Engineering, University of Missouri, 209 Agricultural Engineering Building, Columbia, MO 65211, USA; 2 Department of Biological Systems Engineering, University of Nebraska-Lincoln, Lincoln, NE 68588, USA; yge2@unl.edu (Y.G.); derek.heeren@unl.edu (D.M.H.); cneale@nebraska.edu (C.M.U.N.); 3 School of Natural Resources, University of Nebraska-Lincoln, Lincoln, NE 68588, USA; ewalter-shea1@unl.edu; 4 Daugherty Water for Food Global Institute, University of Nebraska, Lincoln, NE 68588, USA; mmaguire2@unl.edu; 5 Department of Agricultural and Biological Engineering, Pennsylvania State University, University Park, PA 16802, USA; sfi5068@psu.edu

**Keywords:** unmanned aerial systems, wireless communication, crop canopy interference, long-range radios, multi-rotor unmanned aerial system, flight scheduling, data ferry, communication success rate

## Abstract

Agriculture is considered a hotspot for wireless sensor network (WSN) facilities as they could potentially contribute towards improving on-farm management and food crop yields. This study proposes six designs of unmanned aerial system (UAS)-enabled data ferries with the intent of communicating with stationary sensor node stations in maize. Based on selection criteria and constraints, a proposed UAS data ferrying design was shortlisted from which a field experiment was conducted for two growing seasons to investigate the adoptability of the selected design along with an established WSN system. A data ferry platform comprised of a transceiver radio, a mini-laptop, and a battery was constructed and mounted on the UAS. Real-time monitoring of soil and temperature parameters was enabled through the node stations with data retrieved by the UAS data ferrying. The design was validated by establishing communication at different heights (31 m, 61 m, and 122 m) and lateral distances (0 m, 38 m, and 76 m) from the node stations. The communication success rate (CSR) was higher at a height of 31 m and within a lateral distance of 38 m from the node station. Lower communication was accredited to potential interference from the maize canopy and water losses from the maize canopy.

## 1. Introduction

Wireless communication technology has contributed towards the advancement of precision agriculture by providing alternatives to gather and process information [1] which can improve field crop production efficiency and profitability along with natural resources conservation. The technology has contributed to the implementation of wireless sensor networks (WSNs), a compilation of several nodes, with each node being a low-power and low-cost device equipped with one or more sensors, a processor, memory, a power supply, and a transceiver [2]. Each sensor node communicates wirelessly through a communication link and transmits data to a base station or coordinator node via a gateway.

The feasibility of deploying WSNs at low cost has made these systems highly desirable for military, agriculture, sports, medicine, and industry. The potential applications of WSNs as cost-effective processes to improve agricultural resource management have been reported: irrigation management systems [3], farming systems monitoring [4,5,6], pest and disease control [7], controlled use of fertilizers [8], cattle movement monitoring [9], groundwater quality monitoring [10], greenhouse gases monitoring [11], asset tracking [12], and remote control and diagnosis [13,14]. The most promising applications for WSN in precision agriculture are irrigation management, farming monitoring, disease control, and fertilizer accuracy [15]. However, some challenges in WSN applications have been identified such as low battery power, limited computation capability, and sensor node low memory storage [2,16] as well as energy consumption, cost, communication range, optimum deployment schemes, measurement periods, routing protocols, scalability, and fault tolerance [17]. Thus, innovations and creativity in WSN design are needed to be effectively used in agricultural applications.

Unmanned aerial systems (UASs) have been widely used in wireless communication applications because of their high maneuverability and low cost. For an existing WSN, the UAS can be an ideal carrier to form a UAS-based WSN (UWSN). A UAS-based WSN could provide a faster moving speed, longer deployment range, and a relatively longer operating time [18] than WSNs employing other traditional mobile sensor nodes. Recently, the potential scope of a UWSN was identified as a research topic to be investigated for various precision farming applications. Malaver et al. [11] presented the development of a solar-powered UWSN to monitor greenhouse gases (methane and carbon dioxide). Kirichek and Kulik [19] developed analytical and simulation models of a flying ubiquitous sensor network and concluded that such a device is suitable for transferring a small amount of data (transmission rate of 240–280 bits/s) over long distances. Wu et al. [20] investigated the dynamics of uplink and downlink communication using multiple UAS-mounted aerial base stations to serve a ground-based group of users. A rotary-wind UAS-based WSN system for wireless communication was proposed by Zeng et al. [21] and required minimum energy to operate. A remotely piloted unmanned lighter-than-air platform was recently proposed by Gili et al. [22] for the purpose of land use monitoring.

In this study, a UWSN system design is proposed which would comprise hybrid terrestrial surface and sub-surface sensor network stations that communicate with an airborne data ferry deployed on a UAS. Each sensor node station of the network is equipped with three soil water content sensors and one infrared thermometer. The UAS-based data ferrying design is based on criteria and constraints based on the experimental site (research farm) and the nature of the experiment to be conducted. The UAS-based data ferrying system was designed to include wireless technology, a UAS, a radio power source, and memory storage. The major constraints assessing the system were design cost and adoptability.

The design objective was to assemble and test a UAS-enabled data ferrying system that communicates wirelessly with a soil water/crop canopy temperature measurement WSN system with the purpose to monitor soil water content, plant canopy temperature, and air temperature at strategically selected locations across a field in real-time. Six designs based on design objective and constraints were considered and assessed; the best design that met the desired requirements (based on how the system meets the design objective for a field and constraints like that used in this study) was selected and validated in maize over different heights and lateral distances.

## 2. Materials and Methods

### 2.1. Proposed UAS Data Ferrying Designs

#### 2.1.1. Criteria

The wireless communication system was designed to develop a communication channel that sufficiently covers the 50-ha research area at the University of Nebraska’s Eastern Nebraska Research and Extension Center (ENREC) near Mead, Nebraska. The maximum permissible UAS flight height (as per Federal Aviation Administration) is 122 m above ground, thus limiting the testing for successful wireless communication to a 122 m height. Signals between the transceiver (primary) radio and the secondary radio must overlap within their emitted signal footprints to establish communication. Thus, the line of sight (clear communication path) between the transceiver radio and the secondary radio must be considered for a typical US mid-west maize farm (area > 50 ha, i.e., diameter > 800 m). In addition, communication depending on the location of the node station radio could be affected by the vegetative canopy itself, which could cause signal propagation absorption, reflection, attenuation, and scattering [23]. Therefore, the desired range of distance between the radios could be approximately 800 m (considering the size of the farm is 50 ha, and UAS is maneuvering at a height of 122 m) to establish the communication.

The basic scheme is to retrofit the UAS with a wireless transceiver as a data ferry to retrieve datasets from the wireless sensor network (WSN) as the UAS flies across a typical US mid-west maize field. The design should consider a scalable network size scenario where more radios are needed. The transmission time from each node station for the study field should be approximately two minutes to accommodate the battery charge needed by the UAS while it maneuvers over the sensor node stations and downloads data. Thus, the transmission rate is expected to be a few hundred kilobits per second with the minimum data transfer of approximately 2–3 megabits per flight since each sensor node station will record measurements from five sensors frequently.

#### 2.1.2. Constraints

A primary reason for the slow adoption of precision agriculture technology by producers has been the low rate of return on investment. An opportunity to place low-cost sensors and advanced information systems for improving agricultural operations efficiency, while protecting natural resources (non-destructive installation) will add novelty to the overall objective; a low learning curve and low cost will encourage adoption of the wireless technology by producers/agronomists.

#### 2.1.3. Design Parameters and Proposed UAS Data Ferrying Designs

Considering the described design objective, criteria, and constraints, various wireless communication system data ferrying designs were explored based on capabilities, adaptability, and cost of the following factors: (1) wireless technology protocol, (2) UAS, (3) system power source, and (4) data storages.

1. Three wireless technologies designed for industrial/commercial applications that are mostly used for agricultural farm operations were explored for this study: long-range radio (LoRa), ZigBee, and general packet radio service (GPRS). The LoRa protocol was introduced by the LoRa Alliance for the low power and wide-area Internet of Things (IoT) communication associated with the indoor transmission, Pitì et al. [24]. The LoRa gateway can collect data from LoRa nodes to construct the topology of a star network and may communicate with a cloud server over a long communication for high scalability. The LoRa protocol has a wide range of applicability in precision agriculture [6,25,26]. Sensor nodes based on the ZigBee wireless protocol in the agricultural field can communicate with a router up to 100 m. Recent studies have employed ZigBee for precision agriculture [27,28,29] because of its low power consumption, low cost, self-forming characteristics, and suitable communication range. General packet radio service (GPRS)/3G/4G employs packet data (a bit of data that is packaged for transmission) service for GSM-based cellular phones. The GPRS technology communication rates depend on consumer volume where consumers share common communication channels and resources and frequently experience variable delays and throughputs. The sensors could be interfaced to the GPRS system sensor board to obtain and transmit information to the remote server through the GPRS board, which depends on a GSM/GPRS mobile network. Some studies have deployed a GSM/GPRS mobile network for applications in precision agriculture [30,31,32].
2. Fixed-wing and multi-rotor UAS designs were considered as these are mostly used for agricultural operations. Fixed-wing UAS has a longer flight endurance capacity while multi-rotors can provide for stable and easy vertical take-off and landing [33].
3. The power supply to the wireless communication setup on the UAS could be tethered from the UAS or be powered from an external battery source mounted on the UAS.
4. Transmitted data could be stored on the memory storage of the UAS or transmitted to the cloud.

It is essential for the system to be cost-effective and adoptable.

Based on these design parameters, six data ferrying communication systems designs (i.e., a combination of wireless technology protocol, UAS, a power source for the system, and data storages) were assessed:

Design A: ZigBee wireless protocol, fixed-wing UAS, power tethered from UAS, cloud memory storage.

Design B: LoRa wireless protocol, multi-rotor UAS, external power source, memory storage over the UAS.

Design C: GPRS wireless protocol, fixed-wing UAS, external power source, memory storage over the UAS.

Design D: ZigBee wireless protocol, multi-rotor UAS, power tethered from UAS, cloud memory storage.

Design E: LoRa wireless protocol, fixed-wing UAS, power tethered from UAS, cloud memory storage.

Design F: GPRS wireless protocol, multi-rotor, external power source, memory storage over the UAS.

The cost-effectiveness and design adoptability of each design was also evaluated.

#### 2.1.4. Decision Matrix

Proposed designs were assessed based on the design objective for a typical US mid-west maize field (area > 50 ha), the criteria, and the constraints. The proposed designs were evaluated based on an evaluation matrix where points were allocated to the four criteria, and two constraints. The evaluation results are provided in a matrix (Table 1) with scores for each criterion and constraint explained below:

Criteria 1 (wireless technology): The six wireless technology protocol designs were analyzed and assigned points from 1 to 3 (1 being less desirable and 3 being highly desirable). Since the GPRS wireless protocol has a communication range between 1–10 km and a high-power consumption (560 mW), the GPRS was assigned a point value of 1. The ZigBee wireless protocol has a communication range of 100 m and has lower power consumption (36.9 mW) but the line-of-sight between the sensor node and the coordinator node must be available, so the potential for canopy interference could be an issue. Thus, the ZigBee wireless protocol was awarded a point value of 2. Because the LoRa wireless protocol has a long range (5 km) and low power consumption (100 mW), it was rated at 3 points.

Criteria 2 (UAS): The two UAS systems were evaluated and given points from 1 to 2 (with 2 being the more desirable rating). The fixed-wing UAS was rated 1 out of 2 points because of its limitations of low maneuverability, higher cost, and tedious take-off and landing. The multi-rotor UAS was rated 2 points because of its better maneuverability and more controlled take-off and landing as compared to the fixed-wing UAS.

Criteria 3 (power supply): The two types of power supplies studied were given points from 1 to 2. Since the power supply to the wireless communication setup tethered from the UAS could potentially add load on the UAS battery, it was rated 1 out of 2 points. An inexpensive and light external power source mounted on the UAS was determined as a better alternative than tethering the power from UAS since it could sufficiently accommodate the power requirements of the whole system and not add power load to the UAS. Thus, it was assigned 2 points.

Criteria 4 (data storage): Two data storage modes were considered (on-board UAS memory and cloud storage) and awarded points from 1 to 2. Data transmitted over a single flight would be small (around 2 megabits), so cloud storage would not be very helpful in this scenario as it would add unnecessary complexity. Cloud data storage was given 1 out of 2 points, while an on-board UAS memory card was given 2 points.

Constraint 1 (cost): Cost-effectiveness of each system design was evaluated with points ranging from 1–5. Low points were allocated to the design with higher costs. As much as 2 points could be allocated for the cost of the wireless technology protocol, as much as 2 points could be allocated for the UAS cost, and 0 to 1 point for the power source for the wireless communication. The LoRa and ZigBee wireless protocols (allocated 2 points) were low-cost setup systems in comparison to a GPRS wireless protocol (1 point). The fixed-wing UAS is an expensive UAS (allocated 1 point) in comparison to the multi-rotor UAS (2 points), in general. Tethering power from the UAS would be economical (allocated 1 point) in comparison to mounting an external battery source (0 points) on the UAS.

Constraint 2 (adoptability): Adoptability of the design system was determined based on the application of the system by a producer/crop consultant for agronomic decision-making. Points ranging from 1–5 were given to the system based on the adoptability of the system with low points allocated to the design with low adoptability.

‘Design B’ (LoRa wireless protocol, multi-rotor UAS, external power source, memory storage over the UAS) (Table 1) was selected based on the matrix total points and was subsequently investigated as a suitable UAS data ferrying design for operations with a WSN installed in a typical US mid-west maize field.

### 2.2. Field Experiment Description

**Figure 1:**
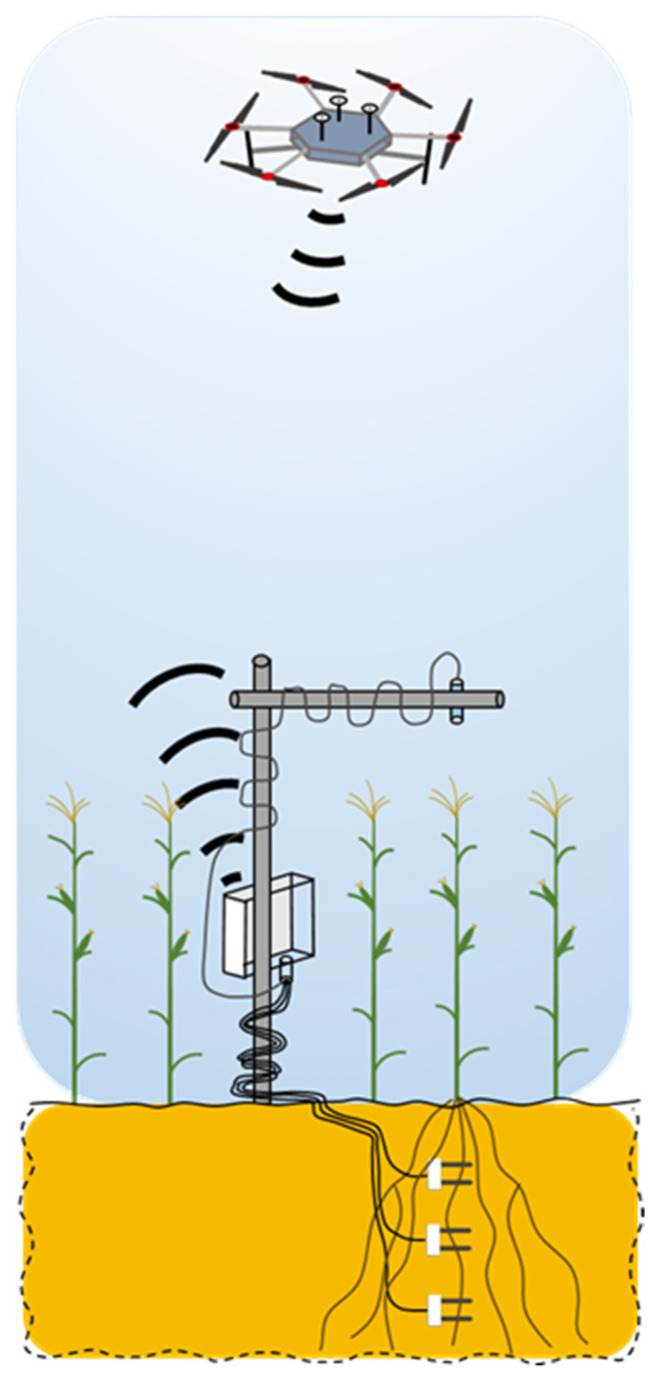
Close-up view of the stationary sensor node station comprising of infrared radiometer and soil water content sensors communicating with an airborne unmanned aerial system-based data ferrying.

A field experiment for testing the proposed UAS data ferrying design was conducted over a nearly 53 ha maize field (typical US mid-west maize field) at the University of Nebraska’s Eastern Nebraska Research and Extension Center (ENREC) near Mead, Nebraska during the 2020 and 2021 growing seasons (Figure 1). Maize was planted in one half of the field and soybean was planted in the other half and was irrigated with a center pivot (Lindsay Corporation Zimmatic 8500). In 2020, the field was planted with soybean in the north half of the field and maize in the southern half. The crops were rotated during the 2021 growing season (soybean in the south, and maize in the north). The study was conducted when the crop was 80% or more coverage and at late crop physiological reproductive stages during 2020 and from mid to late growing season during 2021 (Table 2).

**Figure 2:**
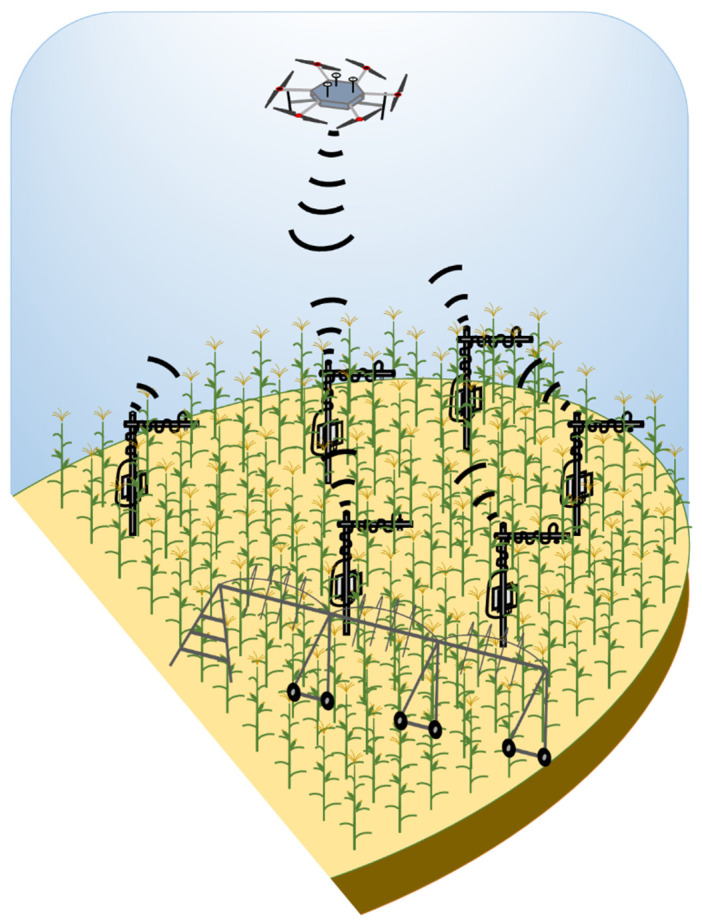
A schematic diagram of airborne based unmanned aerial system data ferry communicating with stationary sensor node stations on the ground installed in maize over a center-pivot irrigated field near Mead, Nebraska.

Stationary sensor node stations comprised of soil water content sensors and infrared radiometer sensors were installed in the maize portion of the field. Four sensor node stations were installed at the beginning of the 2020 growing season and five in 2021. The selected UAS data ferrying design (Design B: LoRa wireless protocol, multi-rotor UAS, external power source, memory storage over the UAS) was constructed and used for wireless communication with the stationary sensor node station; soil moisture and canopy temperature data were to be retrieved from each node station during the UAS flight (Figure 2).

**Figure 3:**
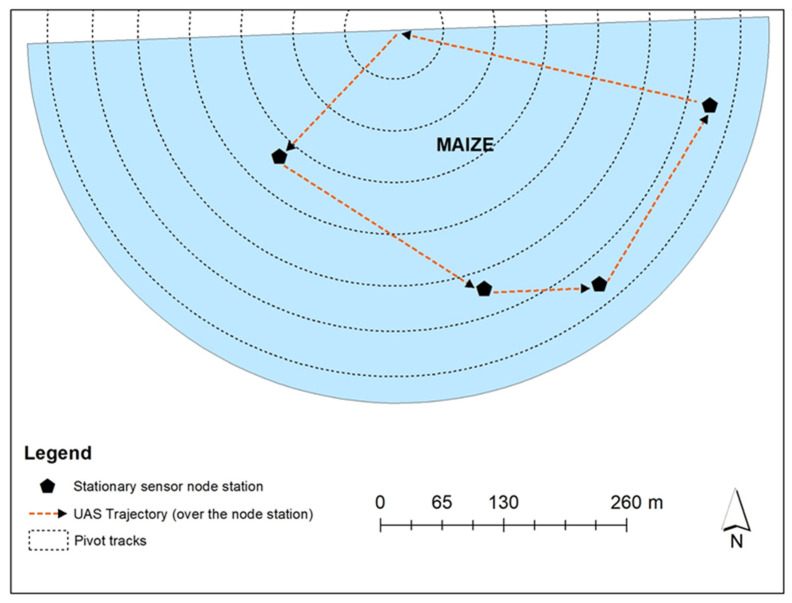
Location of stationary sensor node stations and the trajectory of UAS for four replications during the 2020 growing season. There were three replications of UAS flights that were conducted over the node stations at a height of: (i) 31 m; (ii) 61 m; and (iii) 122 m.

During 2020, communication was attempted with the four replicate node stations at UAS hovering heights of 31 m, 61 m, and 122 m above the node stations for three flight days (Figure 3). The UAS hovered at a lateral distance of 0 m from the node station while the communication attempt was conducted.

**Figure 4:**
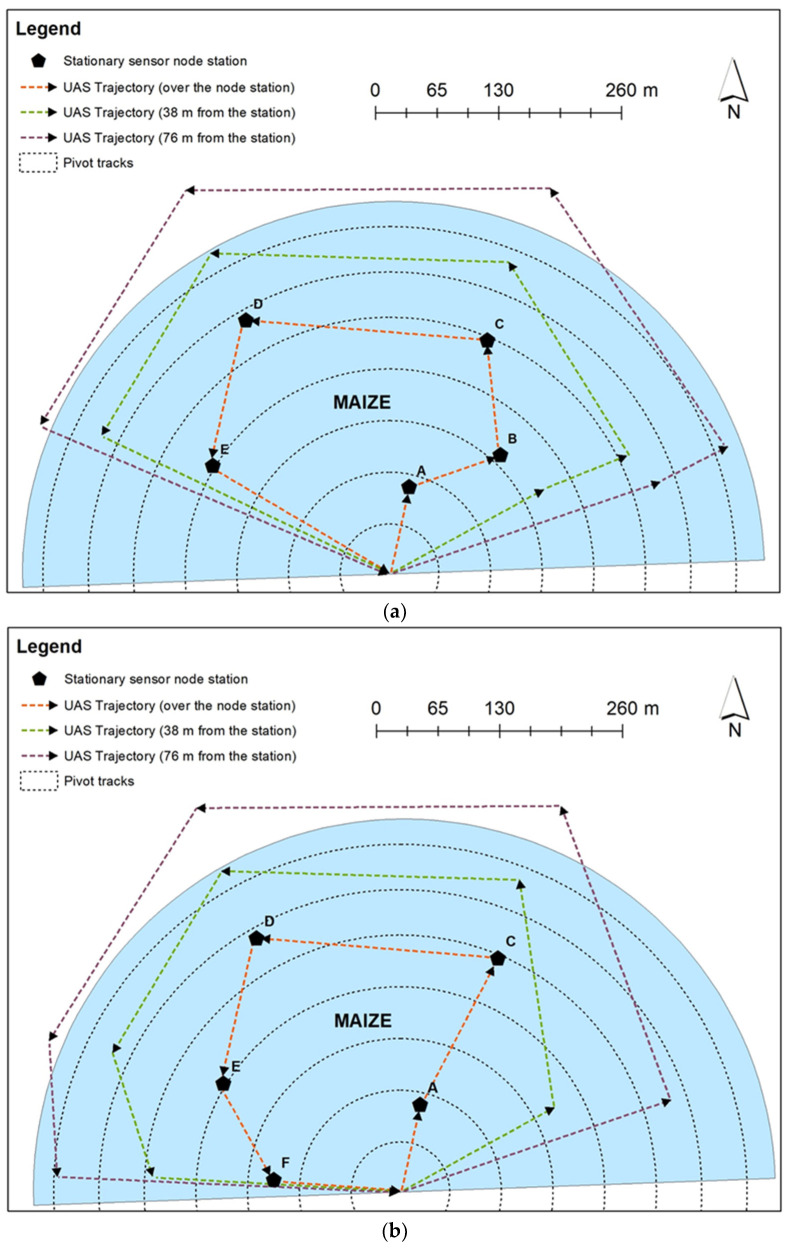
Location of stationary sensor node stations and the trajectory of UAS for five replications during the 2021 growing season: (**a**) before the collapse of the station at point ‘B’, and (**b**) after the installation of the station at point ‘F’. There were three replications of UAS flights: (i) over the station; (ii) 38 m from the station; and (iii) 76 m from the station.

During 2021, communication was attempted with the five replicate node stations at a hovering height of 31 m above the stations and at lateral distances between the UAS and station of 0 m, 38 m, and 76 m during nine flight days (Figure 4). Signal interference due to crop canopy cover was expected to be maximum from the mid to late growing season of maize.

### 2.3. Sensors on the UAS Data Ferry and Node Stations

#### 2.3.1. UAS Data Ferry Sensors

##### RF 450 Radio

RF 450 radio (Campbell Scientific, Logan, UT, USA) is a frequency-hopping, spread-spectrum radio that operates within the 902 to 928 MHz license-free bands designed specifically to work along with Campbell Scientific dataloggers. The radio has a maximum link throughput of 115.2 kbps. The RF 450 radio offers the advantage of high data transfer speeds along with a low current drain. Wireless network communication over long distances (13–60 miles) could be achieved with this radio based on the antenna and the line-of-sight when no objects interfere with the signal.

In a point-to-multipoint network (multi-point network), the transceiver designated as a primary radio can simultaneously communicate with secondary radios. A multi-point network functions with the primary radio (on the UAS in this study) broadcasting its messages to all secondary radios (radios on the node stations); the secondary radios respond to the primary radio when data from the datalogger are received at the data port. A multi-point network was used to collect data from one-to-many dataloggers and report back to one central site.

##### Dipole Omnidirectional Antenna

A 900 MHz, dipole, omnidirectional antenna (Product: 15970, Campbell Scientific, Logan, UT: an indoor antenna with a gain value of 1 dBd [decibels relative to a dipole antenna]) was attached to the secondary RF 450 radios at each node station and housed within the datalogger enclosure located within the canopy.

##### Wave Omnidirectional Antenna

A 900 MHz 0 dBd ½ wave omnidirectional antenna (Product: 14204, Campbell Scientific, Logan, UT, USA) was attached to the primary RF 450 radio mounted on the UAS. The primary antenna has a center frequency of 916 MHz and is equipped with an articulating base that allows the antenna to tilt 90 degrees and rotate 360 degrees, giving flexibility to orient in different directions based on the platform where the antenna is installed (in this study, that is the UAS).

#### 2.3.2. Node Station Sensors

##### CR1000X Datalogger

The CR1000X (Campbell Scientific, Logan, UT, USA) datalogger provides measurement and control for a wide variety of applications because of its reliability and ruggedness. Applications include weather stations, mesonet systems, wind profiling, air quality monitoring, hydrological systems, water quality monitoring, and hydrometeorological stations. The electronics of CR1000X are radiofrequency shielded by a unique sealed, stainless-steel canister. The data logger is equipped with a battery-backed clock that assures accurate timekeeping. The secondary RF 450 radio was attached to the CR1000 datalogger in each node station. See Singh, [34] for details of the operation.

##### GS-1 Soil Water Sensor

The GS-1 sensor (METER Group Inc., Pullman, WA, USA) was used to measure the soil water content (as described in Singh et al. [35]) every 15 min as an average with a sampling frequency of 5s. It is a recently developed capacitance and frequency domain technology-based sensor with a rugged, durable design configured with two parallel waveguide rods (5.2 cm in length).

##### SI-111 Infrared Radiometer

SI-111 infrared radiometer (Apogee Instruments, Inc., Logan, UT, USA) measures emitted infrared radiation (within an atmospheric window of 8–14 μm) from which target surface temperature is remotely determined. The infrared radiometer (also known as an infra-red thermometer or IRTs) monitors the maize field surface temperature continuously every 15 min as an average with a sampling frequency of 5 s [36,37].

#### 2.3.3. Unmanned Aerial System (UAS)

##### Matrice 600 Pro Hexacopter

**Figure 5:**
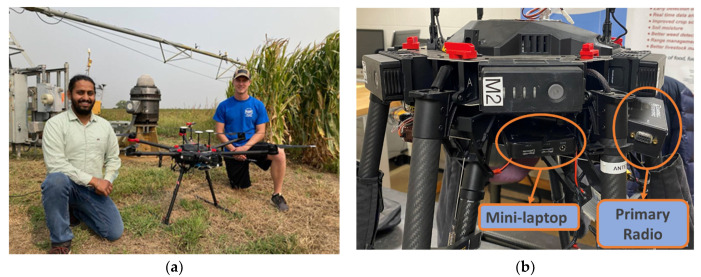
(**a**) The Matrice 600 Pro Hexacopter at the research farm; (**b**) a close-up view of the hexacopter along with the primary radio and the mini laptop.

The DJI Matrice 600 Pro Hexacopter (DJI, Shenzhen, China), a six-rotor flying platform specifically designed for professional aerial photography, industrial, and research applications, was selected as the multi-rotor UAS for this study (Figure 5a,b). The UAS is equipped with dedicated advanced intelligent flight functions, always ensuring safe and stable operation. The Matrice 600 Pro Hexacopter has a maximum takeoff weight of 15.5 kg and a patented battery management system to extend flight time and provide a safe and reliable power supply, suitable for the proposed data ferrying application in fields of approximately 50 ha.

### 2.4. Experiment Design

The transceiver radio (Campbell Scientific’s RF 450 radio) was mounted on the UAS (DJI’s Matrice 600 Pro Hexacopter) along with a mini laptop, a battery source (12 V), and an antenna (Campbell Scientific’s 900 MHz 0 dBd ½ Wave Omnidirectional antenna) (Figure 5a,b). The mini-laptop and the transceiver radio were powered by a 12 V battery source. The mini laptop was connected to the primary radio via a USB cable. The antenna was connected to the radio with the antenna oriented downward. The data retrieval from the datalogger to the secondary radio was scheduled using the ‘Setup’ function of the ‘Loggernet 4.5’ software (see Singh, [34] for details).

**Figure 6:**
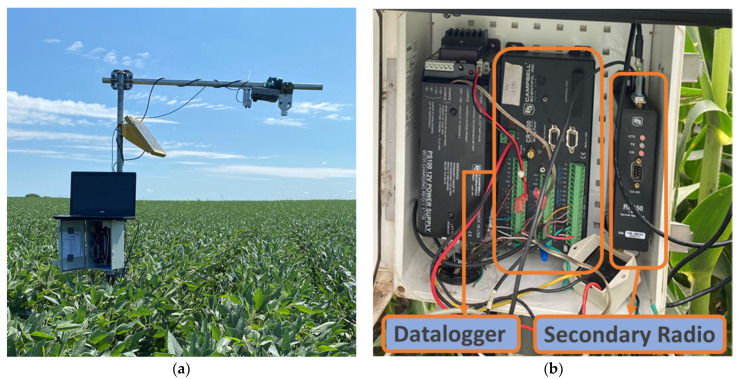
(**a**) Sensor node station comprising of infrared radiometer sensor sensing the crop canopy; (**b**) the layout of the datalogger along with the secondary radio and the power battery source.

Five node stations were installed in the field (A–E) during the 2021 growing season (Figure 4a). One node station collapsed (the galvanized steel pipe on the tripod supporting sensors and instrumentation was corroded and it snapped) in the middle of the growing season (on 7 August 2021) due to the wind shear of a rainstorm; only two UAS flights were conducted with this station. Another node station was installed as a replacement at point ‘F’ in the field (Figure 4b); the remaining seven flights were made after the station at location ‘F’ was installed. The sensor node station comprised of a secondary radio (Campbell Scientific’s RF 450 radio), an antenna (Campbell Scientific’s 900 MHz 1 dBd Omnidirectional antenna), a datalogger (Campbell Scientific’s CR1000X datalogger), a battery source (12 V), soil water content sensors (MeterEnvironment’s GS-1), and an infrared thermometer (IRT) sensor (Apogee Instruments SI-111, Logan, UT, USA). The secondary radio, the antenna, the datalogger, the battery source, and the wires for soil water content sensors and infrared radiometer were enclosed in an enclosure box (Figure 6a,b). The enclosure box was mounted at a height of 1.5 m above ground for stations B, D, and E, and 0.6 m above the ground for stations A and C during the first two experimental trials. During the third experimental trial in 2020, all the enclosure boxes were mounted at a height of 1.5 m above the ground. The location of the enclosure box places the antenna within the canopy when the maize is at full height. The secondary radio was connected to the datalogger via a null modem cable. The soil water content sensors were inserted at a depth of 0.15 m, 0.46 m, and 0.76 m, respectively. The IRT sensor was mounted at a constant height of 1 m above the maize canopy consistently throughout the growing season. All node station sensors were connected to the datalogger. The output from the sensors was recorded at a 5 s sampling frequency and averaged every 15 min.

Soil moisture, canopy temperature, and air temperature data were transmitted from the datalogger through the secondary RF450 radio to the transceiver (primary) RF450 radio mounted on the UAS. Communication success and data transmission and receipt were assessed at different UAS heights (31 m, 61 m, and 122 m above the ground) in 2020, and at different lateral distances of the UAS from the node stations (0 m, 38 m, and 76 m) at a height of 31 m above the ground in 2021.

Communication success was quantified in terms of CSR (in %). The CSR for each treatment and each trial day was calculated as:

(1) $$\mathrm{CSR}\;\left(\mathrm{in}\;\%\right)=\frac{N_{\mathrm{established}}}{N_{\mathrm{attempted}}}\times 100$$

where CSR is communication success rate, N_established_ is the number of replications where the communication was established, and N_attempted_ is the number of replications where communication was attempted.

Wireless communication and data retrieval on the UAS data ferry was scheduled via the UAS-mounted mini laptop. Data retrieval between the UAS mini laptop was scheduled before the UAS flight using the unique PakBus address associated with the CR1000X datalogger of each sensor node station. While the UAS was in the line of sight of a sensor node station, the UAS data ferry transceiver radio (which was connected to the mini laptop) would contact and establish wireless communication with the nearby sensor node station secondary radio. The UAS was scheduled to take one minute to fly from the start point to station ‘A’ where it would establish wireless communication with the sensor node station secondary radio and download data within a minute before moving to the next station (station ‘B’), where it would repeat the contact, wireless communication, and data retrieval protocol for each node station until the UAS returned to the start point (Figure 4a). Given the time required for the UAS to fly from one node station to another (approximately 1 min each) and the time required to retrieve data from a sensor node station when the UAS maneuvers over the station (approximately 1 min), the entire data collection by the UAS data ferry from start to finish took 9 min in 2020 with four node stations and 11 min in 2021 with five node stations.

## 3. Results

Soil moisture and crop canopy temperature data from stationary sensor node stations were transmitted from the secondary radio (which was linked to the node station datalogger) to the UAS data ferry transceiver radio (which was connected to the UAS data ferry mini laptop). The seven-day data file retrieved from each sensor node station was around 0.3–0.4 megabytes (MB) in size and took approximately one minute to retrieve. Each dataset retrieval was comprised of around 10,000 values (soil water content at three depths, crop canopy, and air temperatures were reported once every 15 min). To accommodate data retrieval and the approximate one minute for the UAS to maneuver from one sensor node location to another, the data retrieval from each sensor node station was scheduled two minutes after the previous sensor node station.

Three UAS data ferrying experimental trial events were held during the 2020 growing season (September 2, September 16, and September 30). The UAS flights were conducted at two to three UAS heights (31 m, 61 m, and 122 m), with the four sensor node stations installed in the maize. Communication was attempted during the experimental trial events. The maize crop was at the early denting growth stage on the first experimental trial event/flight day (Table 3). However, for the second and third flight days, the maize was 75% past denting reproductive stage (R5.75), and at physiological maturity. A 100% CSR was observed at 31 m above the ground for all attempts (Table 4) but not for the other UAS heights. Therefore, the 31 m height was used in investigating the effect of crop canopy interference on radio communication along different lateral distances (0 m, 38 m, and 76 m) from the stationary sensor node stations during the 2021 growing season.

**Figure 7:**
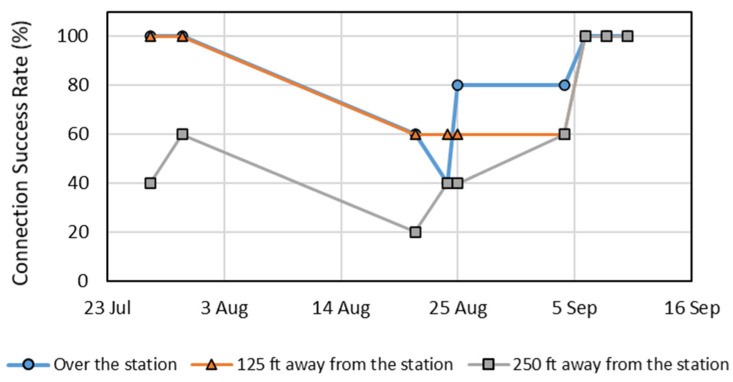
The CSR (in %) for the five replications of secondary radios communicating with the primary radio mounted on the UAS for three treatments during the 2021 growing season. The UAS maneuvered at a height of 31 m above the ground at a lateral distance of: (i) 0 m from (over) the sensor node station; (ii) 38 m from the sensor node station; and (iii) 76 m from the sensor node station.

Nine experimental trial events were conducted during the 2021 growing season; the first two events were conducted before the collapse of the station at point ‘B’ on 7 August 2021 (Figure 4a) in the field, and the remaining seven events were conducted after the installation of the station at point ‘F’ (Figure 4b) in the field. During the first two events, the maize was in the initial physiological reproductive stages (silking and early-blister, i.e., R1 and R2; Table 3), while the maize was in various phases of dent stage R5 (Table 3) for the remaining seven events. The CSR for the first two trials was 100% when the UAS (at a height of 31 m) was directly over the node stations (0 m lateral distance) and 38 m laterally away from node stations; the CSR was within 40–60% when the UAS was 76 m laterally away from node stations (at a height of 31 m) (Figure 7). The next four trials were conducted during the late reproductive stages of maize (early to mid-denting, i.e., R5.1–R5.4; Table 3), and the CSR was within 40–80% when the UAS was over the node stations, 60% when the UAS was 38 m laterally away from the stations, and within 20–60% when the UAS was 76 m laterally away from the node stations. For the last three experimental trial events (around the mid-denting reproductive stage and beyond, i.e., R5.5–R5.6; Table 3), the CSR for all three lateral distances was 100% (Figure 7).

## 4. Discussion

Wireless communication applications in sensor networks in an agriculture setting are still at an early stage of development, and it is already contributing towards the improvement of agricultural management practices. The agricultural sector will be highly benefited by the Internet of Things (IoT) technologies as the management and analysis of IoT data could potentially be used to automate processes, predict situations, and improve agricultural management activities in real-time [38]. In addition, the scope of IoT has increased recently in agricultural activities such as farming, planting, and animal rearing [17,39]. The current study proposes a UAS-enabled data ferrying system that uses narrow band-internet of things technology, i.e., the LoRa (long-range radio) technology, as a means of monitoring water and plant status in an agricultural setting, building on previously published work on the real-time monitoring and calibration of soil water content sensor [35]; and developing inter-relationships between soil water depletion and crop canopy temperature differential [36], and; sensor-based irrigation management of maize and soybean [37]. The LoRa and ZigBee technology offer various advantages such as lower power consumption and data transmission over longer distances in comparison to other wireless technologies or protocols used in agricultural applications, such as WiFi, Bluetooth, GPRS/3G/4G, and SigFox [23]. The comparison indicated that ZigBee and LoRa wireless protocols are more convenient for agricultural applications because of their low power consumption, a suitable communication range for ZigBee, and a long-range communication range for LoRa. For the UAS data ferrying system proposed in the study, LoRa wireless protocol was used considering potential interference from the maize canopy. Similarly, Mahmoud and Mohamad [40] demonstrated that LoRa is a good candidate for low-power and point connectivity for longer distances amongst other wireless communication techniques. Wixted et al. [41] evaluated the performance of LoRa technologies for indoor and outdoor settings, and across physical layers (a mix of newer concrete, glass, and sandstone buildings) wireless and multi-gateway wide area networks and found the technology can provide a reliable link for low-cost remote sensing applications.

The real-time information on soil water and plant canopy temperature status using wireless communication could be highly valuable for farmers who do not have access to weather information due to electricity limitations or limited media access. The low power and LoRa wireless sensor networks can be transmitted to cloud platforms for both private and public networks to facilitate optimal resource utilization and real-time data accessibility from everywhere.

The optimal data transmission rate for the network architecture in this study was 240–480 bits/s, and the LoRa technology’s sizable hardware implementation makes it very suitable for the UAS data ferrying designed in this study. With the proposed data ferrying design (using LoRa wireless protocol, multi-rotor UAS, external power source, memory storage over the UAS), the size of data transmitted from the stationary node station was approximately 4000–5000 bits in one minute. The study by Kirichek and Kulik [19] and Reda et al. [42] confirms the suitability of the LoRa technology.

In this study, commercially available LoRa-based radios were used for wireless communication in a UAS-enabled data ferry WSN (UWSN) in a maize field during the late reproductive growth stages. The CSR for the radios was at 100% when the UAS maneuvered at a height of 31 m above the ground; the CSR was lower when the UAS was at heights of 61 and 122 m. During the mid to late growing season when the maize plants were in the early R5 stages, a lower CSR (20–60%; average 60%) occurred when the lateral distance between the UAS and the stationary sensor node station was at 76 m. The lower CSR was attributed to interference from the maize crop canopy; radio frequencies are attenuated by water [43] and it is assumed that the radio communication has higher interference from moisture in the vegetation. The average maize plant moisture content changes from 60% to 30% at denting stage (R5), and physiological maturity stage (R6), respectively [44]. Eventually, when a substantial amount of water was lost with lower green biomass around the mid-denting stage, the CSR increased (around 100% for all three lateral distance treatments). Antenna placement within the enclosure which is embedded in the canopy could potentially be an issue contributing to the lower CSR during the maize denting stage and needs to be further researched to potentially strengthen communication possibilities.

The UAS data ferrying design recommended, based on the established criteria uses LoRa wireless protocol, multi-rotor UAS, external power source, and memory storage; the measurement protocol recommended within a UWSN using this data ferry is to have the UAS maneuvering at 31 m above the ground and close to the stationary sensor node station (lateral distance of 0 m to 38 m) to establish excellent communication and data retrieval. Thus, a UWSN based on the design proposed here could be used by a producer or farmer to monitor in near real-time soil water and plant status to help farmers make agronomic decisions to increase their agricultural productivity. The proposed approach of UAS enabled data ferrying based wireless communication also could be deployed for public, private, or industrial IoT applications in irrigation management systems, farming systems monitoring, pest and disease control, cattle movement monitoring, groundwater quality monitoring, greenhouse gases monitoring, asset tracking, and remote control and diagnosis.

Since UAS and UAS-related technology have only recently developed and are currently evolving at a fast pace, the technology has not been widely applied for developing UWSN systems. Unexpected problems in this study had to be addressed while developing, troubleshooting, and fine-tuning the technology and procedures used to retrieve data from the maize field. System components such as the connector cable (with thin insulation) connecting the mini-laptop and the radio on the UAS would detach occasionally during the flight, due to air resistance from the wind created while the UAS spinning blades would push the air down during takeoff. The issue was resolved by replacing the initial connector cable with a connector cable with thick insulation that did not detach from the radio as it was heavier and was unaffected by air resistance. Weather elements can take a toll on equipment. Backup equipment and additional replications should be considered in planning for potential setbacks. One of the stationary sensor node stations collapsed (the galvanized steel pipe on the tripod supporting sensors and the instrumentation was possibly corroded and snapped due to the wind shear of a rainstorm in the middle of the growing season (7 August 2021). The station was removed from the study and replaced by a new stationary sensor node station using the radio which was still operable. The new location meant that the data retrieval scheduling on the primary radio had to be revised and ready for the next flight (the flight route and time allocated for maneuvering from one station to another, and data retrieval).

In general, the technology related to the application of UAS as a platform for data ferrying (flying, integrating wireless communication devices, or retrieving datasets while flying) is a rather novel approach with additional work needed to design and evaluate the entire UWSN system (from node stations to UAS based data ferry). While the main purpose of the UWSN system was meant to perform real-time monitoring of soil moisture and crop canopy temperature for the purpose of irrigation management, significant time was spent developing, troubleshooting, and fine-tuning the technology and procedures used to retrieve the datasets. The methodology adopted in this study could be replicated/run on a testing framework such as FlockAI to investigate the result reproducibility of this experiment [45]. Future research should aim to investigate the scalability and adoptability of the proposed data ferrying system to strengthen the applicability of the UAS data ferrying system. The feasibility of mounting additional advanced instrumentation to the existing design of a data ferrying system for simultaneously capturing thermal/multispectral imagery could also be explored as it would aid in efficient irrigation and agronomic decision-making.

## 5. Summary and Conclusions

Six designs of UAS-enabled data ferrying systems with IoT-enabled communication using radios were proposed based on design criteria and constraints. A final design of a data ferry was selected which comprised of a long-range radio wireless communication protocol, an external power source, a multi-rotor UAS, and memory storage. The selected design of the UAS data ferry was tested over a WSN in a maize field in eastern Nebraska during the latter portions of the 2020 and 2021 growing seasons. Each stationary sensor node station installed in maize contained three soil water content sensors, one infrared radiometer, and one secondary radio. CSR at various UAS heights above the stationary nodes (in 2020) and lateral distance (in 2021) from the nodes was investigated. Three experimental trials during the 2020 growing season demonstrated that a 100% CSR occurred when the UAS maneuvered directly over the node station at a height of 31 m above the ground. Henceforth, the UAS was flown at a height of 31 m above the ground during the 2021 growing season to investigate the effect of lateral distance of the UAS data ferrying from the node station on the CSR. For the nine experimental trial events (flight days) during the 2021 growing season, the CSR was higher (80% average) when the UAS was directly over the node station (0 m lateral distance) and at a lateral distance of 38 m from the node station. The CSR was lower when the UAS was 76 m laterally away from the node station (60% average). In general, the lower CSR at higher heights and further distances from the node station was potentially due to the interference from the maize canopy and plant moisture and the location of the antenna in the enclosure; during late reproductive physiological stages when plant water is lower a higher CSR was achieved for all lateral distances as the crop progressed to physiological maturity.

Substantial progress was made in understanding the UAS-based data ferrying system and the potential of the UWSN system to improve agronomic decision making. Future studies to investigate the scalability, adoptability, and optimum deployment scheme (along with mounting an RGB-infrared imaging camera for aid in agronomic decision making, and antenna location) for the proposed data ferrying design could strengthen the applicability of the UWSN system.

## Figures and Tables

**Table 1:** The unmanned aerial systems (UAS) data ferry decision matrix table for six proposed designs based on the four criteria and two constraints with the total points for each design.

	Criteria 1 Wireless Technology	Criteria 2 UAS	Criteria 3 Power Supply	Criteria 4 Data Storage	Constraint 1 Cost	Constraint 2 Adoptability	Total
Design A	2	1	1	1	3	3	11
Design B	3	2	2	2	4	5	18
Design C	1	1	2	2	4	3	13
Design D	2	2	1	1	5	5	16
Design E	3	1	1	1	3	3	12
Design F	1	2	2	2	5	4	16

**Table 2:** The planting date, the observed vegetative and reproductive growth stage dates for maize during the 2020 and 2021 growing seasons.

Maize Growing Season	Planting Date	Vegetative Period	Reproductive Period
2020	11 May	28 May–16 July	17 July–29 September
2021	28 April	14 May–16 July	17 July–26 September

**Table 3:** UAS flight dates and associated maize physiological growth stages during 2020 and 2021 growing seasons.

2020 Growing Season		2021 Growing Season	
Flight Date	Physiological Growth Stage	Flight Date	Physiological Growth Stage
September 2	R5.3	July 27	R1 (silking)
September 16	R5.75	July 30	R2 (early blister)
September 29	R6	August 21	R5.1 (early dent)
		August 24	R5.2
		August 25	R5.25
		September 4	R5.4
		September 6	R5.5
		September 8	R5.55
		September 10	R5.6

**Table 4:** The communication success rate (CSR) (in %) for three treatments (31 m, 61 m, and 122 m above the ground) during the 2020 growing season in maize.

	CSR (in %) during 2020 Growing Season in Maize		
Flight Date	31 m above Ground	61 m above Ground	122 m above Ground
September 2	100%	-	25%
September 16	100%	50%	50%
September 30	100%	75%	75%

## Data Availability

Data is contained within the article. No additional data was created or analyzed in the study.
